# Neurite outgrowth inhibitory levels of organophosphates induce tissue transglutaminase activity in differentiating N2a cells: evidence for covalent adduct formation

**DOI:** 10.1007/s00204-020-02852-w

**Published:** 2020-08-04

**Authors:** Ibtesam S. Almami, Maha A. Aldubayan, Shatha G. Felemban, Najiah Alyamani, Richard Howden, Alexander J. Robinson, Tom D. Z. Pearson, David Boocock, Alanood S. Algarni, A. Christopher Garner, Martin Griffin, Philip L. R. Bonner, Alan J. Hargreaves

**Affiliations:** 1 School of Science and Technology, Nottingham Trent University, Nottingham, NG11 8NS UK; 2 Department of Biology, College of Science, Qassim University, Al-Qassim, Saudi Arabia; 3 Department of Pharmacology and Toxicology, College of Pharmacy, Qassim University, Al-Qassim, Saudi Arabia; 4 Department of Medical Laboratory Science, Fakeeh College for Medical Science, Jeddah, Saudi Arabia; 5 Department of Biology, Faculty of Science, University of Jeddah, Jeddah, Kingdom of Saudi Arabia; 6 Department of Life Sciences, School of Health Sciences, Birmingham City University, City South Campus, Edgbaston, B15 3TN UK; 7 Department of Pharmacology and Toxicology, Faculty of Pharmacy, Umm Al-Qura University, Mekkah, Saudi Arabia; 8 Department of Life and Health Sciences, Aston University, Birmingham, B4 7ET UK

**Keywords:** Organophosphate toxicity, Neurite outgrowth, Covalent adduct, Tissue transglutaminase

## Abstract

Organophosphate compounds (OPs) induce both acute and delayed neurotoxic effects, the latter of which is believed to involve their interaction with proteins other than acetylcholinesterase. However, few OP-binding proteins have been identified that may have a direct role in OP-induced delayed neurotoxicity. Given their ability to disrupt Ca^2+^ homeostasis, a key aim of the current work was to investigate the effects of sub-lethal neurite outgrowth inhibitory levels of OPs on the Ca^2+^-dependent enzyme tissue transglutaminase (TG2). At 1–10 µM, the OPs phenyl saligenin phosphate (PSP) and chlorpyrifos oxon (CPO) had no effect cell viability but induced concentration-dependent decreases in neurite outgrowth in differentiating N2a neuroblastoma cells. The activity of TG2 increased in cell lysates of differentiating cells exposed for 24 h to PSP and chlorpyrifos oxon CPO (10 µM), as determined by biotin-cadaverine incorporation assays. Exposure to both OPs (3 and/or 10 µM) also enhanced in situ incorporation of the membrane permeable substrate biotin-X-cadaverine, as indicated by Western blot analysis of treated cell lysates probed with ExtrAvidin peroxidase and fluorescence microscopy of cell monolayers incubated with FITC-streptavidin. Both OPs (10 µM) stimulated the activity of human and mouse recombinant TG2 and covalent labelling of TG2 with dansylamine-labelled PSP was demonstrated by fluorescence imaging following SDS-PAGE. A number of TG2 substrates were tentatively identified by mass spectrometry, including cytoskeletal proteins, chaperones and proteins involved protein synthesis and gene regulation. We propose that the elevated TG2 activity observed is due to the formation of a novel covalent adduct between TG2 and OPs.

## Introduction

It is well established that many commercially available organophosphorous compounds (OPs) used as oil additives (e.g., tricresyl phosphate; TCP) and insecticides (e.g., chlorpyrifos; CPF), inhibit the activities of serine hydrolases such as acetyl and butyryl cholinesterase (Aldridge 1954; Chambers and Levi 1992; Gupta 2006; Kamanyire and Karralliede 2004). However, they are also capable of inducing delayed neurodegenerative and/or developmental effects through covalent interactions with non-cholinesterase protein targets (Ray and Richards 2001; Hargreaves 2012; Sánchez-Santed et al. 2016).

For example, TCP induces a neurodegenerative condition known as OP-induced delayed neuropathy (OPIDN), which was associated with a major outbreak of *Ginger Jake* poisoning during the years of prohibition in the USA (Bishop and Stewart 1930; Zeligs 1938). The clinical signs of OPIDN do not appear until up to several weeks following exposure and are characterised by wrist drop and a flaccid paralysis (Abou-Donia and Lapadula 1990; Hargreaves 2012). The onset of OPIDN is preceded by a number of early molecular lesions, such as irreversible inhibition of neuropathy target esterase (NTE), disrupted Ca^2+^ homeostasis, proteolytic degradation and altered phosphorylation of cytoskeletal proteins (Hargreaves 2012) in the central and peripheral nervous systems. It is known that T*O*CP (i.e., the *ortho* isomer of TCP) is the main inducer of OPIDN by TCP formulations (Abou-Donia and Lapadula 1990; Harris et al. 1997) following its metabolic conversion into saligenin cyclic-*o*-tolyl phosphate (SC*O*TP), which is a more potent inhibitor of NTE but, unlike OPs used as pesticides, a weak inhibitor of AChE (Lotti and Johnson 1978). A number of cell culture studies have shown that phenyl saligenin phosphate (PSP), a structural analogue of SC*O*TP, inhibits the outgrowth of neurites in differentiating neuroblastoma cells in association with complete inhibition of NTE, increased activation of ERK 1/2, and transient hyperphosphorylation followed by a reduction in the protein levels of neurofilament heavy chain (Hargreaves et al. 2006).

On the other hand, the main acute toxicity target of CPF, after its conversion to chlorpyrifos oxon (CPO) by cytochrome P450-mediated desulphuration, is AChE, the inhibition of which leads to the accumulation of acetylcholine and, potentially, subsequent cholinergic stress (Saunders et al. 2012; Hargreaves 2012). However, CPF also exhibits delayed neurotoxic effects in developing rodent brains, following exposure at doses causing non-acute inhibition of AChE activity but able to inhibit protein and DNA synthesis, thus affecting the normal development of the brain (Grandjean and Landrigan 2014; Lee et al. 2015).

It has been shown that CPF and its metabolites induce neurodevelopmental toxicity in embryonic stem cell models of neural differentiation, as indicated by characteristic patterns of disruption of differentiation marker gene expression, via a mechanism that is dependent on the expression of NTE but not on the inhibition of its esterase activity (Estevan et al. 2014; Sogorb et al. 2016). In vitro studies have also shown that CPF and CPO inhibit neurite outgrowth (Howard et al. 2005; Sachana et al. 2001, 2008; Yang et al. 2008; Flaskos et al. 2011), proliferation and DNA synthesis in neuronal and glial cell lines (Garcia et al. 2001; Qiao et al. 2001). CPF interferes with the AChE transduction cascade (Yang et al. 2008), and with transcriptional events by affecting AP-1 and SP1 nuclear transcription factors that are involved in neuronal cell differentiation (Garcia et al. 2001; Crumpton et al. 2000), and with neurotransmission (Slotkin and Seidler 2007). CPF also affects the adenylate cyclase signalling pathway (Garcia et al. 2001) that controls the formation of cAMP, which acts as an important switch signal to activate cell differentiation (Cooper and Hausman 2004).

Glial cells exposed to CPF exhibit an increase in ROS formation (Saulsbury et al. 2009). The creation of ROS is not associated with direct chemical actions; rather, it involves the effects of CPF on cell metabolism (Crumpton et al. 2000). Studies on cultured cells have indicated that elevated levels of ROS can disrupt Ca^2+^ homeostasis (Xu et al. 2011), which could consequently interfere with the regulation of Ca^2+^ dependent proteins.

Of interest to the present study are the transglutaminases (TGs), which comprise a family of Ca^2+^ dependent enzymes that catalyse the post-translational modification of proteins (Lorand and Graham 2003; Bergamini et al. 2011). Tissue TG-associated enzymatic functions, such as the covalent cross-linking of proteins and amine incorporation into proteins, are associated with tissue stabilization and with the prevention of and protection from bodily injury. However, TG2 activity is inappropriately activated in some neuropathologies (Kim et al. 2002), thus making it a potential therapeutic target.

Tissue transglutaminase (TG2) plays an important role in several processes that occur in normal neural tissues. For example, it is involved in the plasticity and stabilization of the synapse in the CNS by formation of protease-resistant protein crosslinks (Festoff et al. 2001). It is also involved in the suppression of catecholamine release from neurons, generating a negative feedback that will also prevent an excessive release of transmitters. Tissue TG expression and transamidase activity are required for neurite outgrowth in differentiating neuroblastoma cells (Tucholski et al. 2001) and rat cerebellar granule neurons (Mahoney et al. 2000). It was suggested that this effect involved TG2-mediated increases in cAMP production due to enhanced activation of cAMP response element binding protein (CREB) (Tucholski and Johnson 2003), a transcription factor that modulates synaptic plasticity, cell survival and cell differentiation (Duman et al. 2000). The presence of TG2 and its cross-linking activity have also been detected in the growth cones of neurites produced by cultured cerebellar granule neurons, in which several substrates for TG2 have been identified (Sugitani et al. 2006).

As TG2 is a Ca^2+^ dependent enzyme, its activity could be disrupted by altered Ca^2+^ homeostasis, as well as by elevated levels of ROS, phenomena which are known to be induced by OPs (Giordano et al. 2007; Ientile et al. 2007). Preliminary studies by our group have shown that exposure to PSP results in disruption of TG2 activity monitored in lysates from mitotic N2a and HepG2 cells after 24 h exposure. It is also interesting to note that TG2 protein levels suffered a decrease in PSP-treated N2a cells but increased in HepG2 cells, suggesting that the changes in the protein levels could have been responsible for the differences in the observed effects (Harris et al. 2009a).

Given the emerging role of TG2 in neurite outgrowth and neural differentiation, either through its expression or its catalytic activities, coupled with its potential involvement in neurotoxicity, the aim of this study was to explore the modulation of TG2 activity by exposure to the OPs PSP and CPO in differentiating mouse N2a neuroblastoma cells.

## Materials and methods

Unless otherwise specified, all reagents were purchased from Sigma Aldrich Company Ltd^.^ (Poole, UK). Dulbecco’s modified Eagle’s medium (DMEM) with 4.5 g/L glucose and 2 mM L-glutamine was obtained from Lonza (Viviers, Belgium). Cell culture plastic ware and other cell culture reagents were purchased from Scientific Laboratory Supplies (SLS, Wilford, UK). The N2a and C6 cell lines were from obtained from ATCC and ECACC, respectively.

### Maintenance and differentiation of N2a cells

Cells were grown as monolayers in T75 flasks with 20 ml growth medium (high glucose DMEM supplemented with 10% v/v foetal bovine serum (FBS), 2 mM L-glutamine, penicillin (100 units/ml) and streptomycin (100 μg/ml)), using a humidified incubator at 37 °C and an atmosphere of 5% CO_2_/95% air. Cells were sub-cultured on reaching approximately 80–90% confluence. For differentiation experiments, cells were seeded at 50,000 cells/ml into 24-well plates (25,000 cells/well), T25 (500,000 cells) or T75 flasks (2 million cells) in growth medium and allowed 24 h recovery before the induction of cell differentiation in the absence and presence of OPs. For this, stock solutions of OPs were dissolved in dimethyl sulphoxide (DMSO) and diluted in pre-warmed serum-free medium containing 0.3 mM dibutyryl cAMP, immediately before use, to final concentrations of 1–10 µM. The final concentration of DMSO was 0.5% (v/v), which was also added to control cells. In some experiments, as described below, to confirm the involvement of TG2, cells were pre-incubated for 1 h with the irreversible TG2 specific inhibitor Z-DON-Val-Pro-Leu-OMe (ZDON; Zedira, GmbH, Germany), prior to the induction of cell differentiation. This was added at a final concentration of 100 μM (Almami et al. 2014).

### Measurement of cell viability and neurite outgrowth

To determine the effects of OPs on cell viability, cells were seeded in T25 flasks (500,000 cells in 10 ml growth medium) and cultured for 24 h prior to the induction of differentiation in the absence and presence of OPs (1–10 µM), as described above. Following exposure, the cells were detached and viable cell counts determined by Trypan Blue exclusion using a TC-20 automated cell counter (BioRad, Hemel Hempstead, UK) after a 1:1 dilution with Trypan Blue solution (0.4% v/v; BioRad, Hemel Hempstead, UK).

Neurite outgrowth was monitored by high content analysis (HCA) of monolayers immunofluorescently stained with monoclonal antibody 2G10, which recognises βIII-tubulin, using an ImageXpress^MICRO^ imaging system (Molecular Devices, Oxford, UK), as described previously (Sindi et al. 2016). Significant outgrowth was recorded for any cell with at least one stained neurite longer than 5 µm in length.

### Preparation of cell lysates

After the appropriate treatments, cell monolayers were rinsed twice with 2 ml of chilled PBS, lysed with 500 μl of ice-cold lysis buffer consisting of 50 mM Tris buffer pH 8.0, 0.5% (w/v) sodium deoxycholate, 0.1% (v/v) protease inhibitor cocktail III (Millipore, Watford, UK) and 1% (v/v) HALT™ Phosphatase Inhibitor Cocktail (1:100; Thermo Fisher Scientific, Paisley, UK). Cell lysates were scraped and clarified by centrifugation at 4 °C for 20 min at 14 000 × *g*. Supernatants were collected and stored at − 20 °C. Protein concentration was measured by the bicinchoninic acid (BCA) protein assay, based on the method of Stoscheck (1990) using a kit from Sigma Aldrich (Poole, UK). For Western blot analysis, cell monolayers were rinsed as above, then lysed with 500 μl of 0.1% (w/v) sodium dodecyl sulphate (SDS) in PBS (pre-heated to 100 °C), after which the suspension was heated at 100 °C for 5 min and then centrifuged at 100,000 *g* for 30 min, before supernatants were collected and stored at − 20 °C.

### Measurement of TG2-mediated amine incorporation in cell lysates

Biotin-cadaverine assays were performed on cell lysates by the method of Slaughter et al. (1992) as modified by Lilley et al. (1998). For this, 96-well microtitre plates (Nunc Maxisorp; SLS, Wilford, UK) were coated overnight at 4 °C with N',N'dimethylcasein (10 mg/ml in 100 mM Tris buffer pH 8.0; 250 µl/well). The plate was washed twice with distilled water and blocked with 250 μl of 3% (w/v) BSA in 0.1 M Tris buffer, pH 8.0 and incubated for 30 min at 37 °C. The plate was washed twice before adding 150 μl of 100 mM Tris buffer, pH 8.0, containing 225 μM biotin-cadaverine (Fisher Scientific, Loughborough, UK), with 2 mM β-mercaptoethanol and either 6.67 mM calcium chloride to activate or 13.3 mM EDTA to inactivate TG2. The reaction was started by the addition of 50 μl of samples or positive control (50 ng Guinea pig liver TG2) and negative control (Tris buffer). After incubation for 1 h at 37 °C, plates were washed as before, after which 200 μl of 100 mM Tris buffer pH 8.0, containing horseradish peroxidase (HRP)-conjugated ExtrAvidin™ (1:500 dilution) were added to each well and the plate incubated at 37 °C for 45 min then washed as before. The plates were developed by incubation at room temperature for 15 min with 200 μl of freshly made developing buffer (7.5 μg/ml 3, 3′, 5, 5′ tetramethylbenzidine and 0.0005% (v/v) H_2_O_2_ in 100 mM sodium acetate pH 6.0. The reaction was terminated by adding 50 μl of 5.0 M sulphuric acid and the absorbance read at 450 nm. One unit of TG2 was defined as a change in absorbance at 450 nm of 1.0 per hour. Each experiment was performed in triplicate.

### Measurement of TG2-mediated amine incorporation in situ

N2a cells were seeded into ibidi µ-slide cell culture treated 8-well chamber slides (Thistle Scientific, Glasgow, UK), at 50,000 cells/ml, adding 300 μl of medium per well, and cultured for 24 h in growth medium. Cells were then treated for 1 h with or without the TG2 inhibitors Z-DON (100 µM) and induced to differentiate as described above by the addition of differentiation medium containing either 3 or 10 µM PSP or 10 µM CPO for 24 h. During the last 4 h of differentiation, 1 mM biotin-X-cadaverine (5-(((*N*-(biotinoyl)amino)hexanoyl)amino) pentylamine), trifluoroacetic acid salt; Fisher Scientific, Loughborough, UK) was added. Following treatment, cells were fixed with 3.7% (w/v) paraformaldehyde in PBS for 15 min at room temperature, before being permeabilised with 0.1% (v/v) Triton-X100 in PBS for 15 min at room temperature. Each of the above steps was followed by three 5 min washes with PBS. Cell monolayers were blocked by incubation with 3% (w/v) BSA in PBS for 1 h at room temperature, after which biotin-X-cadaverine incorporation was detected by incubation with fluorescein isothiocyanate (FITC)-conjugated ExtrAvidin® (green fluorescence) diluted 1:200 in blocking buffer, for 1 h followed by 3 PBS washes, as described above. Nuclei were stained with blue fluorescence (DAPI; Invitrogen, UK) and images viewed in an epifluorescence microscope.

### In situ labelling for gel based detection of proteins serving as substrates for TG2

For this, N2a cell monolayers were induced to differentiate in T75 flasks (2 million cells per flask) in the presence and absence of OPs, adding 1 mM biotin-X-cadaverine (Fisher Scientific, Loughborough, UK) for the last 4 h of differentiation. Cell monolayers were lysed as described by Singh et al. (1995), after which proteins in cell lysates were subjected to SDS-PAGE (40 μg/well) and transferred to nitrocellulose membranes (Laemmli, 1970; Towbin et al. 1979). The biotin-cadaverine labelled proteins were detected by probing blots with ExtrAvidin^®^-HRP and visualized by enhanced chemiluminescence (ECL). In some experiments, biotin-cadaverine labelled proteins were enriched using CaptAvidin™ agarose (Life Technologies Ltd., Paisley, UK) prior to SDS-PAGE, as described by Almami et al. (2014).

### Proteomic analysis of biotin-cadaverine labelled proteins

Following labelling with 1 mM biotin-X-cadaverine, biotin-cadaverine labelled proteins were extracted, as described above. The proteins labelled with biotin- cadaverine were purified using CaptAvidin™ agarose (Life Technologies Ltd., Paisley, UK) prior to polyacrylamide gel electrophoresis in the presence of SDS (SDS-PAGE) (Almami 2014). Protein bands were visualized using InstantBlue protein stain. CaptAvidin-captured proteins with molecular weights matching bands showing increased labelling on gels of lysates from cells differentiated in the presence of PSP that was attenuated by pre-treatment with ZDON, were excised and subjected to in gel digestion, as described by Aldubayan et al. (2017). Briefly, gel bands were excised from the gel and cut into small pieces using a sterile scalpel. The gel pieces were de-stained with freshly prepared 100% methanol and 50 mM (NH_4_)HCO_3_ (1:1 v/v). Protein bands were subjected to reduction and alkylation, by sequential incubation with 25 mM DTT in 50 mM (NH_4_)HCO_3_ followed by 55 mM iodoacetamide in 50 mM (NH_4_)HCO_3_ solution. The gel pieces were then processed for trypsin digestion (Trypsin Gold; Promega, Southampton, UK) over night at 37 °C. The reaction was terminated by adding 0.5% (v/v) trifluroacetic acid (TFA) to a final concentration of 0.5% (v/v) and digested proteins were recovered in the supernatant following centrifugation.

### Mass spectrometry and protein identification

The full profiling of enriched biotin-cadaverine proteins was achieved by LC–MS/MS. Briefly, samples were injected by trap-elute (4 µL) using an Eksigent 425 LC system (trap: YMC triart C_18_ 0.3 × 5 mm, 300 µm ID. Analytical column: YMC Triart C_18_ 150 × 0.3 mm, 3 µm, 5 µL/min) into a SCIEX TripleTOF 6600 mass spectrometer in Information Dependent Acquisition (IDA, top 30) mode via gradient elution (mobile phase A: 0.1% formic acid; B: acetonitrile in 0.1% formic acid) over 87 min (2% to 30% B over 68 min; 40% B at 72 min followed by column wash and re-equilibration) (Aldiss et al. (2019). Mass spectrometry raw data were processed using ProteinPilot 5.02 (SCIEX, Warrington, UK) against the SwissProt mouse database (January 2019) and 1% FDR protein cut-off applied.

### Statistical analysis

Quantitative data are expressed as mean ± SEM for a minimum of 3 independent experiments. Statistical significance of differences was determined by one-way or two-way ANOVA (as appropriate), followed by Tukey's post hoc test, using GraphPad Prism^®^ software. A significant difference was assumed if *P* < 0.05.

## Results

### Effects of PSP on N2a cell viability and neurite outgrowth

**Fig. 1:**
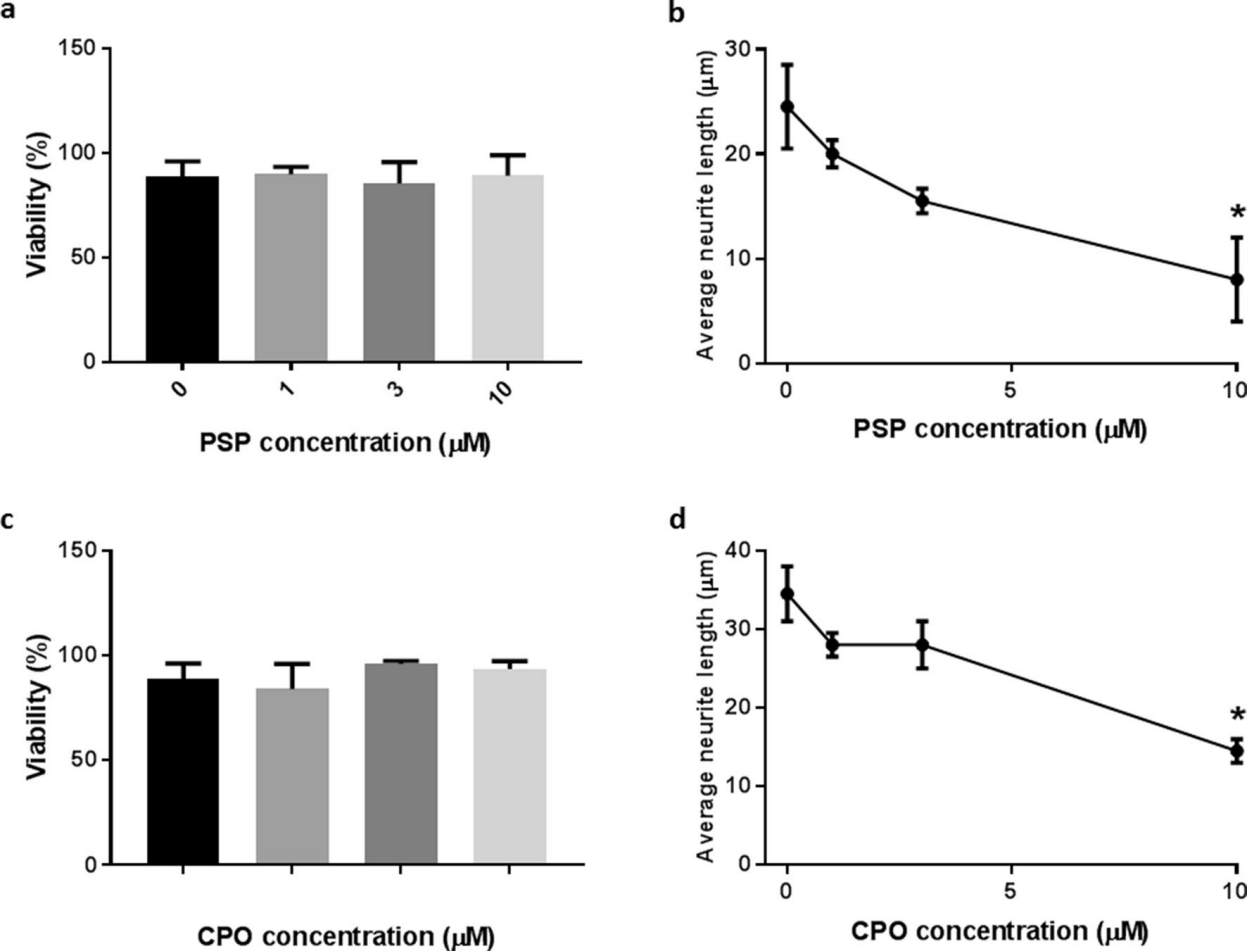
Effects of PSP and CPO on the viability and differentiation of N2a cells. N2a cells were induced to differentiate for 24 h in the absence and presence of PSP (**a**, **b**) and CPO (**c**, **d**) at 1–10 µM. They were then either detached and incubated with Trypan Blue solution to determine cell viability (**a**, **c**) or fixed and stained by indirect immunofluorescence with anti-βIII tubulin antibody for determination of neurite outgrowth by HCA (**c**, **d**), as described in “Materials and methods”. Data are expressed as mean ± SEM for at least 3 independent experiments. Asterisks indicate the level of statistical significance of differences (***P < 0.001, **P < 0.01, *P < 0.05)

The ability of PSP to affect the viability and differentiation of N2a cells was monitored by Trypan Blue exclusion and HCA, respectively. Over the concentration range tested (1–10 µM) PSP had no statistically significant effect on the exclusion of Trypan Blue (Fig. 1a). It did, however, induce a concentration-dependent reduction in average neurite length, as indicated by HCA of differentiating cell monolayers stained by indirect immunofluorescence using anti-βIII tubulin antibody (Fig. 1b). Similar trends were observed in the case of treatment with CPO (Fig. 1c, d).

### Effect of PSP on TG2 activity in differentiating N2a cells

**Fig. 2:**
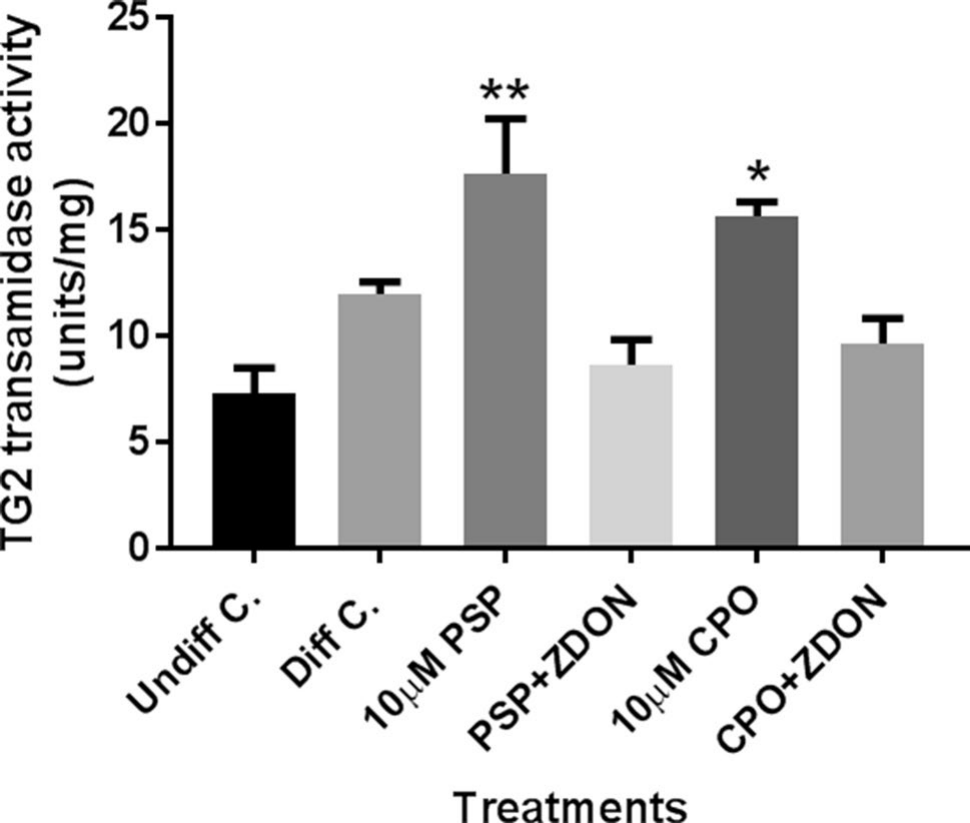
Effects of PSP and CPO on TG2 activity in cell lysates from differentiating N2a cells. Mitotic N2a cells were pre-treated in the absence or presence of the TG2 inhibitor Z-DON (100 μM) for 1 h and then subjected to differentiation for 24 h in serum-free medium containing 0.3 mM dibutyryl cAMP, in the absence or presence of PSP or CPO (both at 10 µM). Cell lysates were subjected to biotin-cadaverine incorporation assays, as described in “Materials and methods”. Data points represent the mean ± SEM of TG2 specific activity from 3 independent experiments. Data analysis was performed using one-way ANOVA with the Tukey post hoc test. Asterisks indicate the level of statistical significance of differences from the control (***P < 0.001, **P < 0.01, *P < 0.05)

For initial studies of the ability of PSP to disrupt TG2 activity, lysates prepared from N2a cells induced to differentiate in the presence and absence of 10 μM PSP or CPO in serum-free medium containing dibutyryl cAMP, were subjected to assays for the incorporation of biotin cadaverine, which acts as the acyl-acceptor for TG2. The data shown in Fig. 2 show a slight increase in TG2 activity on cell differentiation and a statistically significant increase in activity on differentiation in the presence of PSP compared to control undifferentiated cells. In the case of PSP-treated cells that were co-treated with the TG2 inhibitor ZDON there was a significant decrease in amine incorporation compared to cells treated with PSP alone. Similar results were obtained for CPO exposure.

**Fig. 3:**
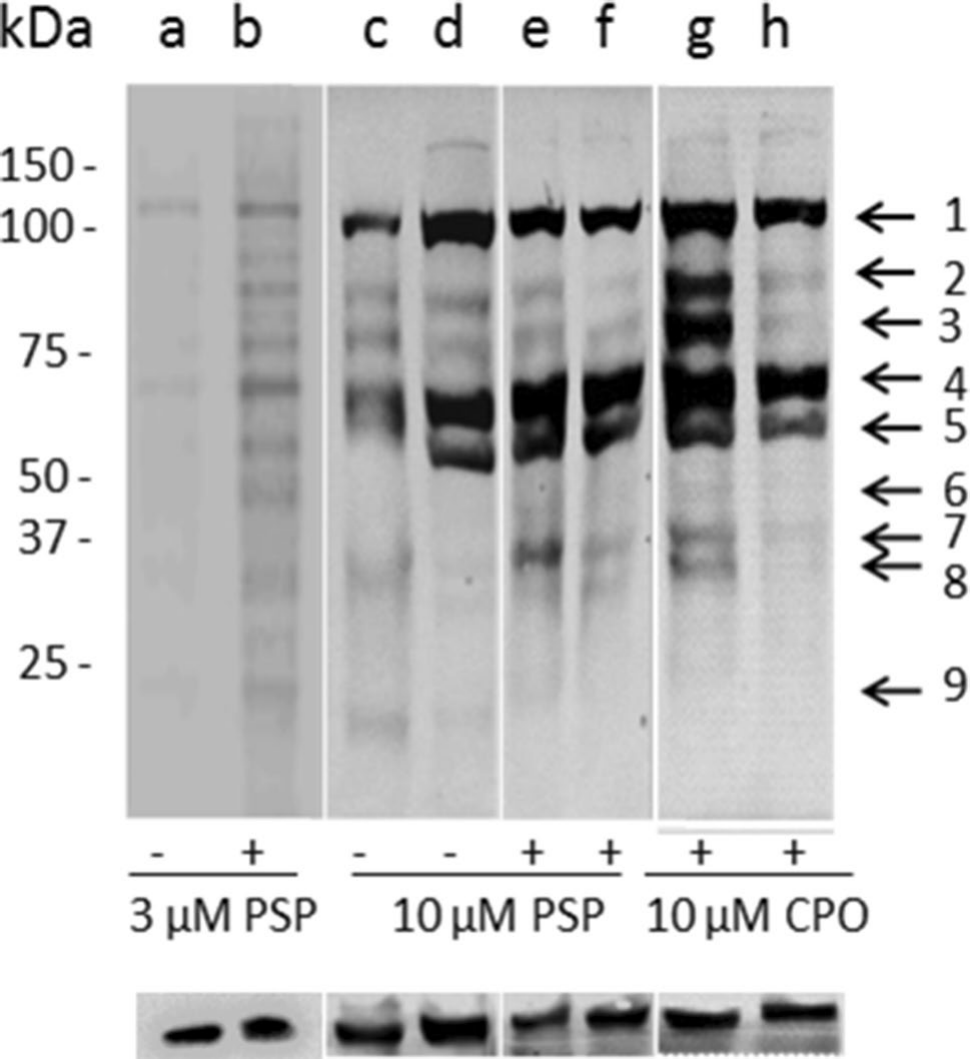
Western blot analysis of the effects of PSP on in *situ* TG activity in differentiating N2a cells. Cells were pre-incubated for 1 h with (**f**, **h**) or without (**a**–**e** and **g**) the TG2 inhibitor Z-DON (100 μM) before the induction of cell differentiation for 24 h in the absence and presence of 3 μM (**a**, **b**) or 10 μM PSP and CPO (**c**–**h**). Well (**c**) contained non-differentiated (i.e., mitotic) cell lysate protein. For the last 4 h of treatments, 1 mM biotin-X-cadaverine was added. The total protein extract was resolved by SDS-PAGE (40 μg per lane) and transferred onto nitrocellulose membranes. Biotin-cadaverine labelled proteins were detected using ExtrAvidin^®^-HRP. Samples were subsequently analysed on a separate blot using an anti-GAPDH antibody as a control for protein loading (lower panel). The arrows point to biotin-ExtrAvidin-labelled proteins that show increased labelling following exposure to PSP or CPO that was attenuated by pre-treatment with ZDON

**Fig. 4:**
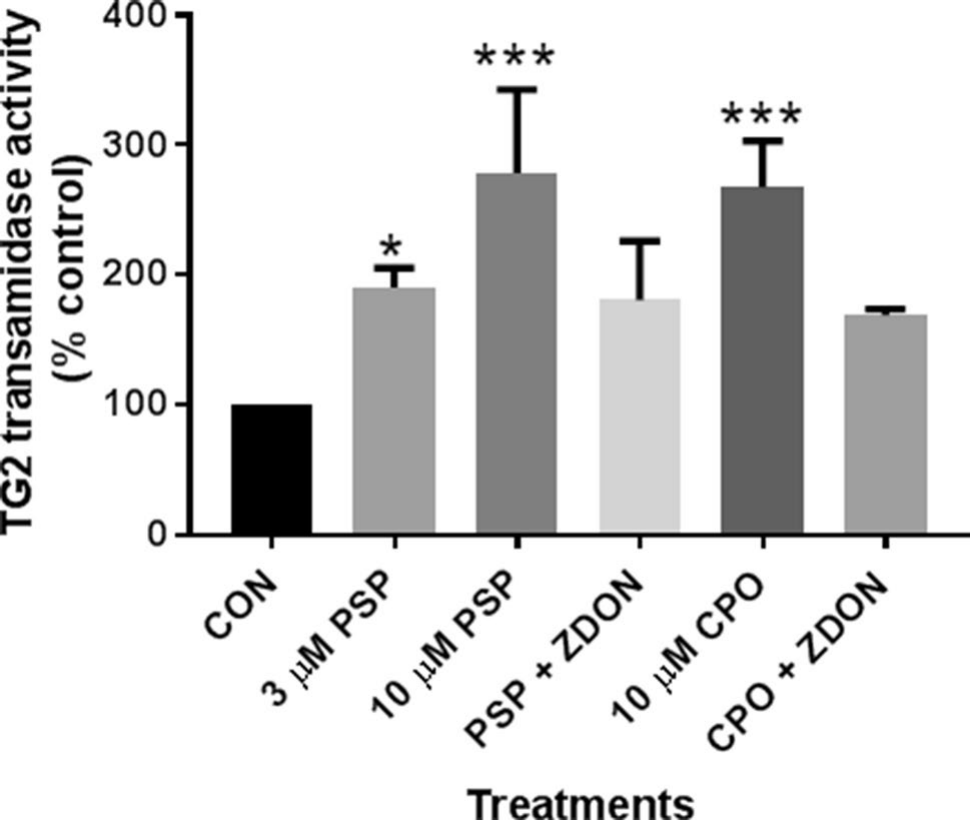
Densitometric analysis of biotin-cadaverine incorporation into proteins detected on Western blots of cell lysates from differentiating N2a cells. Following in situ labelling of differentiating N2a cells with biotin-X-cadaverine, Western blots of cell lysates (shown in Fig. 3) were probed with ExtrAvidin^®^-HRP, as described in “Materials and methods”. Densitometric analysis of each lane (total labelled protein) was carried out using GelQuant software and the data are expressed as the percentage of basal TG2 substrate protein levels ± SEM after GAPDH normalization. Data analysis was performed using one-way ANOVA with the Tukey post hoc test. Asterisks indicate the level of statistical significance of differences from the differentiating cell control ***P < 0.001, **P < 0.01, *P < 0.05

**Fig. 5:**
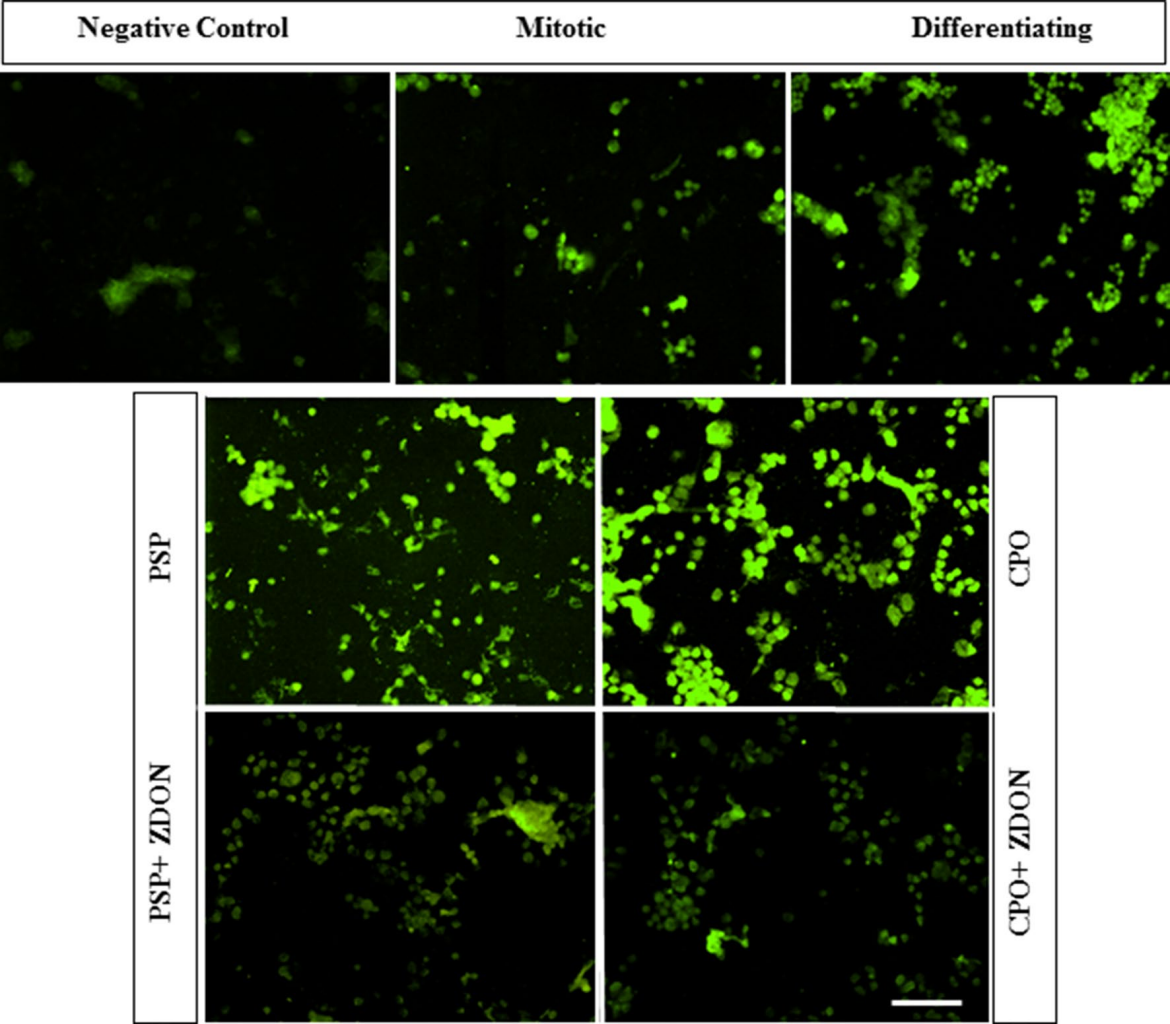
Measurement of the effects of organophosphates on in situ TG2 activity in differentiating N2a cells using Extravidin^®^-FITC. Cells were seeded in chamber slides and incubated with biotin-X-cadaverine after treatment with or without 10 µM PSP, 10 µM CPO and ZDON, followed by Extravidin^®^-FITC (green), as described in “Materials and methods”. Shown are typical images from a representative experiment (one of three). Images show typical fields of N2a cells, treated for 1 h with or without the TG2 inhibitor Z-DON (100 μM) before the induction of differentaition in the presence and absence of 10 μM PSP or CPO for 24 h. For the last 4 h of treatments, 1 mM biotin-X-cadaverine was added, except for one set of untreated cells used as a negative control with no biotin-cadaverine. Nuclei were stained with DAPI (blue). The original magnification of the images was 20×. Bar represents 200 µm

It was then of interest to determine whether the same trend would be observed for TG2 activity in situ. As shown in Fig. 3, there was an apparent increase in the level of biotin-X-cadaverine incorporation into selected protein bands on Western blots of lysates prepared from N2a cells induced to differentiate in the absence and presence of PSP (3, 10 µM) compared to non-OP-treated control cells, with molecular weights ranging from ~ 25 to 150 kDa, as indicated by horizontal arrows (Fig. 3). The corresponding bands had labelling similar to that of controls when cells were co-treated with the TG2 inhibitor ZDON (100 µM). Similar results were observed for lysates prepared from differentiating N2a cells exposed to 10 µM CPO (Fig. 3). As shown in Fig. 4, densitometric quantification of whole lanes showed a statistically significant increase in overall band intensity on the developed blots from lysates of OP-treated cells compared to the non-OP-treated controls. These biotin-cadaverine labelled proteins showed reduced staining on western blots of lysates from cells that were incubated in the presence of both OP and the TG2 inhibitor ZDON, the activity returning to basal levels. In situ labelling was also monitored by fluorescence microscopy using FITC-labelled ExtrAvidin™, for which a qualitatively similar pattern of staining was observed, as shown in Fig. 5.

### Direct effects of organophosphates on human and mouse recombinant TG 2 activity

**Fig. 6:**
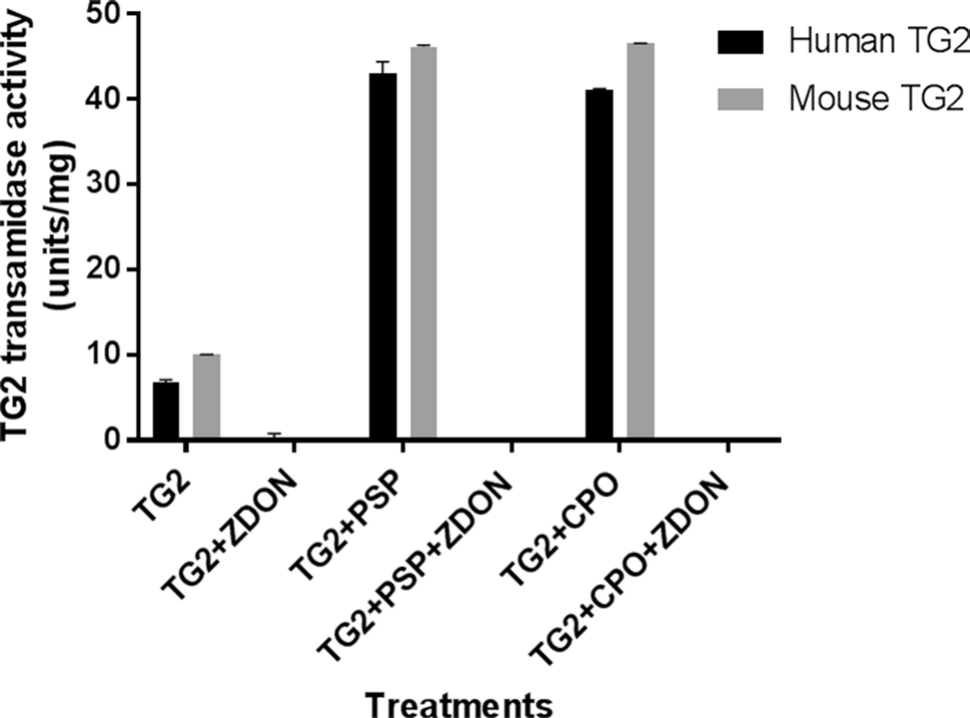
Direct effects of organophosphates on recombinant TG 2 activity. TG2-mediated transamidase activity of human and mouse recombinant TG2 was determined using the biotin-cadaverine incorporation assay as described in “Materials and methods”. Data points represent the mean specific activity ± SEM. Data analysis was performed using two-way ANOVA followed by the Dunnett’s post hoc comparison test, as indicated. Asterisks indicate the statistical significance of differences (***P < 0.001, **P < 0.01, *P < 0.05)

**Fig. 7:**
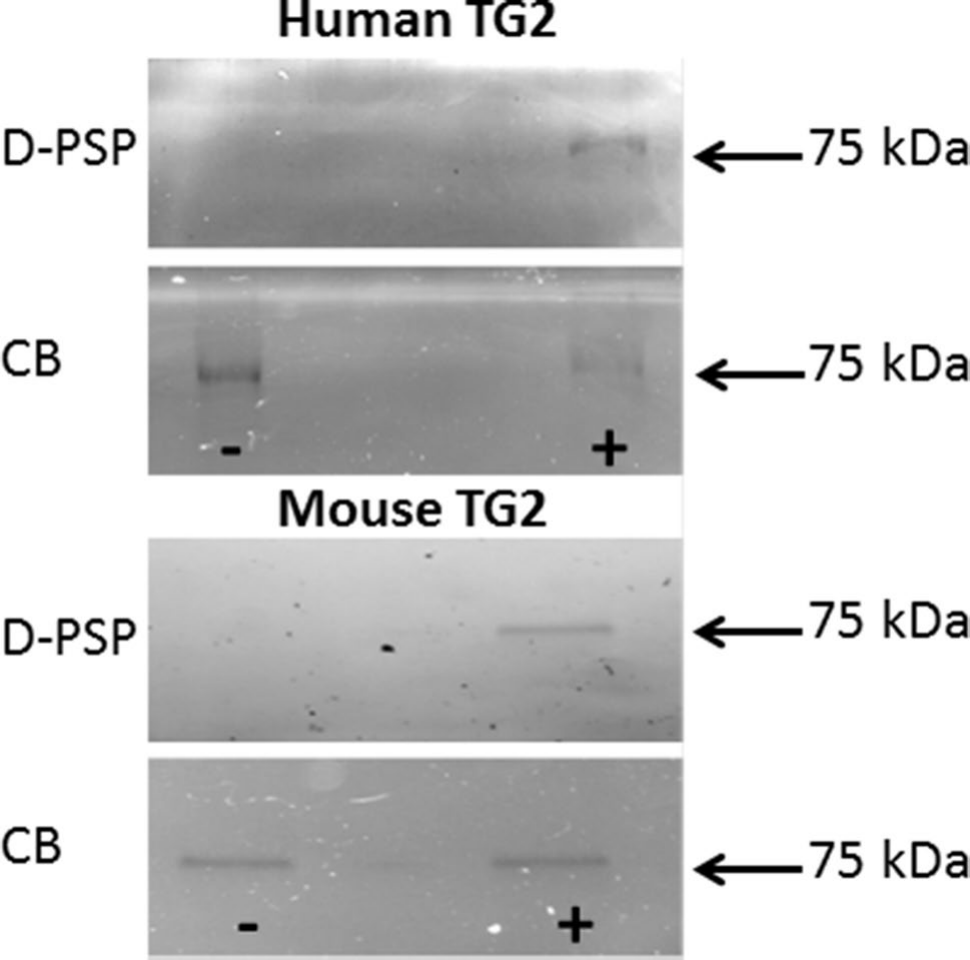
Formation of covalent adducts between TG2 and PSP. Covalent labelling of human or mouse recombinant TG2 with dansylamine-labelled PSP (D-PSP) was detected in a fluorescence imager after SDS-PAGE (upper panels) and Instant Blue staining (CB) of total protein in the same samples (lower panels), as indicated

To study the possibility of a direct effect of PSP on TG2 activity, human and mouse recombinant TG2 were assayed in the absence and presence of 10 µM PSP or CPO using the biotin-cadaverine incorporation assay (Fig. 6). The data obtained indicated a significant OP-induced increase in recombinant TG2 activity. All activity was blocked by co-treatment with 100 μM ZDON. To determine whether this reflected covalent adduct formation between TG2 and PSP, recombinant TG2 was incubated with a dansylamine-labelled analogue of PSP, which had previously been used to identify PSP adducts in cardiomyocyte-like cells (Felamban et al. 2015). The gel images in Fig. 7 show that both human and mouse recombinant TG2 incubated with dansylamine-labelled PSP were detectable as fluorescent bands after separation by SDS-PAGE.

### Effects of PSP on the intracellular distribution TG2 and α-tubulin

**Fig. 8:**
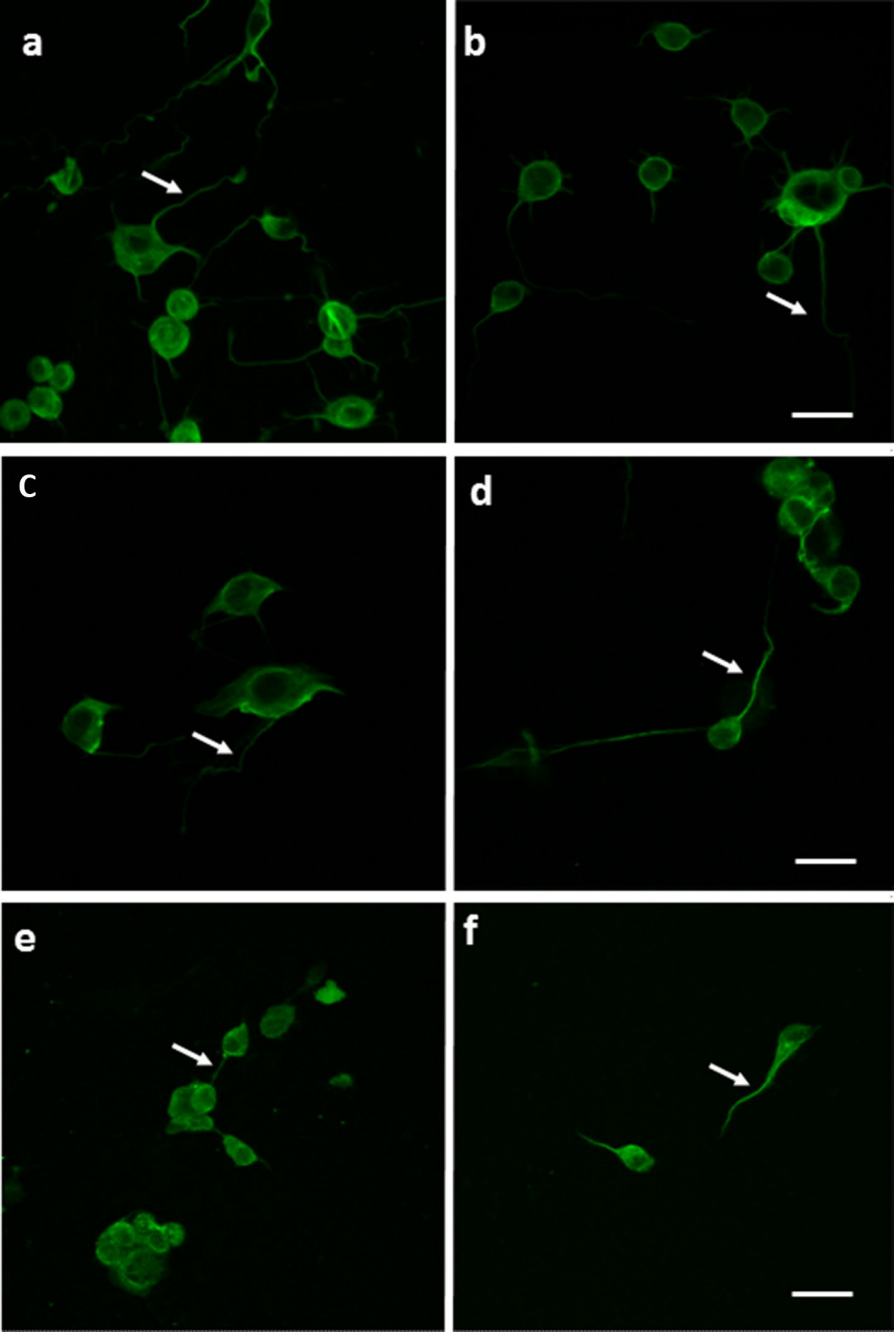
Effects of PSP on the distribution of TG2 and α-tubulin in differentiating N2a cells. N2a cells were induced to differentiate in chamber slides for 24 h in the presence and absence of 3 μM PSP. Cells were then fixed and permeabilised as detailed in “Materials and methods”. Fixed monolayers were probed with monoclonal anti-α-tubulin (clone B512) (**a**, **b**), and two monoclonal antibodies to TG2: clones ID10 (**c**, **d**) and CUB7402 (**e**, **f**). Scale bar represents 20 μm. Arrows indicate typical neurites

It was of further interest to determine whether exposure to OPs affected the intracellular distribution of TG2. As shown in Fig. 8a, b, immunofluorescence staining with a monoclonal antibody that recognises α-tubulin revealed a similar staining pattern with respect to relative intensity of neurite/cell body staining in control and PSP-treated cells. By contrast the relative intensity of staining of neurite: cell body staining with two different anti-TG2 antibodies was higher in PSP-treated cells than in the controls (Fig. 8c–f).

### Identification of protein substrates of TG2-mediated transamidase activity

Prior to identification of TG2 substrates, biotin-cadaverine labelled proteins were enriched using CaptAvidin™ agarose (Almami et al. 2014). Biotin-cadaverine labelled TG2 substrate proteins captured by CaptAvidin™ beads were separated by SDS-PAGE and proteins revealed by staining with Coomassie Brilliant Blue. The protein bands corresponding in molecular weight to those showing activation and inhibition of TG2-mediated biotin-cadaverine incorporation activity were excised from gels (arrows in Fig. 3). The proteins were then extracted from gel pieces and subjected to trypsin digestion after reduction and alkylation, as described in “Materials and methods”. Digested samples were then analysed by LC–MS/MS, as a result of which multiple proteins were identified within each Coomassie-stained band. Proteins with at least 3 peptide matches following ProteinPilot searching against the SwissProt mouse database are listed in Table 1. Proteins that were identified as potential TG2 substrates ranged in molecular weight from approximately 30 to 150 kDa. These included cytoskeletal proteins (e.g., vimentin, peripherin, lamin B1, tubulin and actin), chaperone and folding proteins (e.g., HSPs 60, 70 and 90), Ca^2+^ and phospholipid binding proteins (e.g., matrin 3), mitochondrial proteins (e.g., MICOS complex subunit mic60, Cytochrome b-c1 complex subunit 2 and ATP-citrate synthase), in addition to several other proteins involved in the regulation of protein synthesis (e.g., elongation factors 1-alpha 1, 1-alpha 2 1-gamma and various ribosomal proteins), gene expression (e.g., various isoforms of histone H1), and metabolism (e.g., GAPDH).

According to the TRANSDAB database (https://genomics.dote.hu/wiki/index.php/), some of the identified proteins were already reported as TG2 substrates (Csősz et al. 2009), but they have not as yet been identified as such in differentiating N2a cells though we have identified some as TG2 substrates in H9c2 cardiomyocyte-like cells. Those previously identified by our group include heat shock protein HSP 90-beta, Stress-70 cognate 71 kDa and HSP 60 kDa, isoforms of actin and tubulin, myosin-9, elongation factor 1-alpha 1, tropomyosin, vimentin, histones and heterogeneous nuclear ribonucleoprotein A1 (Almami et al. 2014; Vyas et al. 2016, 2017).

**Table 1:** Identification of TG 2 substrates by mass spectrometry

Band	% Coverage	Accession number (s)	Proteins identified as potential TG2 substrates	Peptide matches	Approx mass (Da)
1	6	Q8VEK3	Heterogeneous nuclear ribonucleoprotein U	6	87,918
	4	Q8K310	Matrin-3	3	94,630
	2.6	Q91V92	ATP-citrate synthase	3	119,728
2	18.4	P58252	Elongation factor 2	14	95,314
3	20.3	P11499	Heat shock protein HSP 90-beta	15	83,281
	7	Q8CAQ8	MICOS complex subunit Mic60	5	83,900
4	30.9	P38647	Stress-70 protein, mitochondrial (GRP75)	26	73,680
	31.7	P63017	Heat shock cognate 71 kDa protein	25	70,871
	13.6	Q9D0E1	Heterogeneous nuclear ribonucleoprotein M	10	77,649
	16.5	P03975	IgE-binding protein	8	62,747
	13.3	P14733	Lamin B1	8	66,786
	11	P20029	78 kDa glucose-regulated protein	6	72,422
	6.7	Q99MN1	Lysine–tRNA ligase	4	67,840
	4.5	P26041	Moesin	3	67,767
5	27.2	P63038	60 kDa heat shock protein, mitochondrial	16	60,955
	15.7	Q60864	Stress-induced-phosphoprotein 1	8	62,582
	15.1, 10.2, 9.2, 8.6, 7.4, 6.9	P61979, Q8R081, P02535, 61,414, P19001, P08730	Heterogeneous nuclear ribonucleoproteins K and L	7-Mar	44,542—63,964
6	25.7	P99024	Tubulin beta-5 chain	13	49,671
	22.8	P20152	Vimentin	10	53,688
	10.5	Q9D8E6	60S ribosomal protein L4	6	47,154
	10.5, 10.4, 10.4, 8.3, 8.7	P68373, P68369, P05213, P68368, and P05214	Tubulin alpha-1C, 1A, 1B, 4A and 3 chains	6, 6, 6, 5, 4	49,909/50,136/50,152/49,924/49,960
	11.5, 9.0	P56480, Q03265	ATP synthase subunits β and α, mitochondrial	5, 4	56,300/59,753
	5.4	P09405	Nucleolin	4	76,723
	7.5	P03975	IgE-binding protein	4	62,747
	8.6	Q922R8	Protein disulphide-isomerase A6	3	48,100
	6.1	P15331	Peripherin	3	54,268
7	38.3	P17182	Alpha-enolase	18	47,141
	15.8	P03975	IgE-binding protein	7	62,747
	14.2	Q9D8N0	Elongation factor 1-gamma	5	50,061
	8.8	P20152	Vimentin	4	53,688
	11.5, 11.5	P62631, P10126	Elongation factor 1-alpha 2 and 1-alpha-1	4, 4	50,454/50,114
	8.2, 8.3	Q9ERD7, P99024	Tubulin beta-3 and 5 chains	3, 3	50,419/49,671
8	22.8	P20152	Vimentin	10	53,688
	17.8	P03975	IgE-binding protein	8	62,747
	19.2, 19.2	P63260, P60710	Actin, cytoplasmic 1 and 2	7, 7	41,793/41,737
	17.6	Q8VE37	Regulator of chromosome condensation	6	44,931
	11.6	Q91VM5	RNA binding motif protein, X-linked-like-1	4	42,162
	11.5	Q9WV02	RNA-binding motif protein, X chromosome	4	42,301
	8.9	Q9D8N0	Elongation factor 1-gamma	4	50,061
	11.5	Q9DB77	Cytochrome b-c1 complex subunit 2, mitochondrial	5	48,235
	8.1	O35685	Nuclear migration protein nudC	3	38,358
9	33.9	P16858	Glyceraldehyde-3-phosphate dehydrogenase	18	35,810
	23.1, 18.4, 30.6	P43277, P15864, P43274	Histones H1.3, H1.2 and H1.4	8, 7, 8	22,100/21,267/21,977
	29.3	P14148	60S ribosomal protein L7	8	31,420
	28.3	P43276	Histone H1.5	7	22,576
	20.8	P97351	40S ribosomal protein S3a	5	29,885
	13.5	P70372	ELAV-like protein 1	4	36,169
	10.2	P25444	40S ribosomal protein S2	3	31,231

Biotin-cadaverine labelled TG2 substrate proteins captured by captavidin beads were separated by SDS-PAGE and the 9 main bands showing increased TG2-mediated labelling were excised and analysed by mass spectrometry. Data shown are for abundant proteins showing a minimum of 3 peptide matches determined with 95% confidence levels. Molecular weight (MW) is also indicated in Da, according to values published on UniProt. As discussed, some of these are potentially novel TG2 substrates not currently listed on the TRANSDAB database (https://genomics.dote.hu/wiki/index.php/) of Csősz et al. (2009) or identified as potential substrates in our own previous work (Almami et al. 2014; Vyas et al. 2016, 2017)

## Discussion

The results presented in this study show that sub-lethal neurite inhibitory concentrations of PSP, an OP associated with the induction of OPIDN, induced elevated levels of TG2-mediated transamidase activity, as determined by assays performed on cell lysates prepared following treatment and by in situ labelling with the membrane permeable substrate biotin-X-cadaverine. The fact that OP- induced increases in transamidase activity in differentiated cells were significantly attenuated by ZDON provided further conformation that TG2 was involved.

The possibility that this might be due to a direct effect of PSP on TG2 was confirmed in assays using mouse and human recombinant TG2, which also showed elevated levels of biotin-cadaverine incorporation in the presence of PSP. This suggested that TG2 could be a novel PSP binding protein, which was subsequently confirmed by the observation of a fluorescent TG2 band following incubation with dansylamine-labelled PSP and separation by SDS-PAGE, indicating the formation of a novel covalent adduct with both mouse and human recombinant TG2.

The fact that similar effects on TG2 activity were observed for the oxon metabolite of the organophosphorothioate CPF (i.e., CPO), suggests that other OPs may also interact directly with TG2. Given the known importance of TG2 in neurite outgrowth (Perry et al. 1995; Mahoney et al. 2000; Tucholski et al. 2001, 2003) an important morphological determinant of neuronal cell differentiation during development and also in nerve regeneration, and the observation of TG2 upregulation in neurodegenerative diseases (Kim et al. 2002; Grosso and Mouradian 2012), our findings suggests that TG2 represents a new and highly relevant non-cholinesterase protein target for OP-binding. It is possible that OP adduction induces the acquisition of a toxic property in TG2, such as that proposed for NTE in OPIDN (Glynn 2000). Alternatively, the changes observed may reflect a protective effect of TG2 upregulation. Indeed, the enrichment of anti-TG2 immunofluorescence staining in surviving neurites in PSP-treated cells, compared to those of control N2a cells, is indicative of a redistribution of TG2, which is consistent with this possibility.

To further investigate the consequences of TG2 upregulation, it was of interest to identify the potential substrates of TG2 in OP-induced inhibition of neurite outgrowth. Analysis of bands of interest in CaptAvidin-captured proteins separated by SDS-PAGE allowed the identification of a number of known and novel potential substrates, none of which had been previously identified as substrates in N2a cells. These could be grouped into several categories based on function, including cytoskeletal proteins, chaperones and proteins involved in gene expression and/or protein synthesis.

For example, 78 kDa glucose-regulated protein Hspa5 chaperone is a quality control protein the endoplasmic reticulum lumen, involved in the correct folding of proteins and degradation of misfolded proteins (Weng et al 2011). It also plays a key role in neurological disorders, acting as a stress sensor, and in embryonic development of the central nervous system (Zhang et al. 2007). It also has an essential role in protection from neuronal apoptosis (Wang et al. 2010). Indeed, a proteomic analysis study conducted by our group before has demonstrated upregulation of 78 kDa glucose-regulated protein in N2a neuroblastoma cells following exposure to the organophosphorous compound diazinon (Harris et al. 2009b). This protein has been already identified as a TG2 substrate in H9c2 cardiomyocyte-like cells (Almami 2014). Whether its modification by TG2- mediated transamidation enhances or attenuates its activity remains to be determined.

Interestingly, an IgE-binding protein was identified in CaptAvidin-captured proteins as a novel protein substrate for TG2. This may reflect the fact IgE receptors have been detected on the surface of neuronal cells and are thought to be involved in a variety of inflammatory responses of sensory neurones to IgE-antigen complexes (Andoh and Kuraishi 2004; Van der Klejj et al. 2010; Liu et al. 2017). This finding is consistent with the possibility that TG2-mediated amine incorporation may be involved in the modulation of such processes.

Several of the other substrates identified in CaptAvidin-captured protein extracts are involved in the regulation of protein synthesis and gene expression, including a number of histones, elongation factors and RNA-binding proteins, etc. Similar types of proteins have been identified as TG2 substrates in studies of adenosine receptor stimulation in cardiomyocyte-like cells (Almami et al. 2014; Vyas et al. 2016, 2017). The nuclear matrix protein matrin 3, on the other hand, is a potentially novel TG2 substrate; it is modulated by Ca^2+^/calmodulin binding, cleaved by caspases and is involved in the control of mRNA stability (Valencia et al. 2007; Salton et al. 2011). Mutations in the matrin 3 gene are linked with familial forms of amyotrophic lateral sclerosis, suggesting an important role in neuroprotection (Johnson et al. 2014). It may be that TG2-mediated amine incorporation can modulate the activity of these groups of proteins during OP-exposure and that this is partly responsible for changes observed in gene expression and protein levels in previous studies on developmental neurotoxicity in brain and cultured cell models (Garcia et al. 2001; Qiao et al. 2001; Howard et al. 2005; Sachana et al. 2001, 2008; Yang et al. 2008; Flaskos et al. 2011; Grandjean and Landrigan 2014; Lee et al. 2015).

Actin and tubulin are the core components of microfilaments and microtubules, respectively, and are significantly expressed in nerve tissue and differentiated neuroblastoma cell lines (Oh et al. 2006). Two isoforms of both actin and tubulin have been identified as TG2 substrates by us previously, following stimulation of adenosine receptors in H9c2 cells (Vyas et al. 2016, 2017) and are well established TG2 substrates. A covalent interaction of CPO with tubulin beta-5 was also observed in extracts from mouse brain (Jiang et al. 2010) suggesting that it is an important target in OP-induced delayed neurotoxicity. The beta-5 tubulin isoform was previously identified as a substrate for TG2 in cardiomyocyte-like cells (Vyas et al. 2016, 2017), although other isoforms of both alpha and beta tubulin are also recognised substrates on the online database (https://genomics.dote.hu/wiki/index.php/). The identification of vimentin and peripherin as substrates suggests that proteins in all 3 cytoskeleton networks (i.e., microtubules, microfilaments and intermediate filaments) are targeted by TG2 in OP-treated cells.

Upregulation of Stip1 is correlated with neuroprotection during ischemic insult; this protein is released from astrocytes and it causes increases in intracellular Ca^2+^ levels by activation of the alpha-7-nicotinic ACh receptor (Beraldo et al. 2013). Stip1, along with HSP 90-beta, stress-70 cognate 71 kDa and HSP 60 are chaperone proteins involved in cytoprotection, modulation of protein folding and transport, and have been shown to be up-regulated after a toxic insult by the OP nerve agent sarin in mouse brain (Chaubey et al. 2017). The level of Stip1 also increased in the brain following ischaemia, inducing neuroprotective signals that rescue the cell from apoptosis (Beraldo et al. 2015). It would be of value to carry out further work to determine the effects of increased amine incorporation on the biochemical properties and functions of this and the other proteins identified as potential substrates of TG2 in OP-treated differentiating N2a cells.

In conclusion, under conditions that cause inhibition of neurite outgrowth, OPs are capable of binding covalently to TG2 and activating TG2-mediated transamidase activity, targeting a number of proteins essential for the integrity of neurites and others involved in the regulation of gene expression, protein synthesis and protein folding.

It could be that OP adduct formation reduces the Ca^2+^ requirement for TG2 activation in situ and/or that it binds to a regulatory domain that exerts an allosteric effect on the active site. Indeed, characterisation of the OP-binding sites on TG2 and their role in TG2 modulation will help to determine the mechanism involved. Further work will also help to determine the precise role of TG2 and its modified protein substrate in OP-induced inhibition of neurite outgrowth.
